# Periodical Urticaria

**Published:** 1874-02-01

**Authors:** J. Schneck

**Affiliations:** Mount Carmel, Ill.


### PERIODICAL URTICARIA.

TWO CASES FROM THE PRACTICE OF J. SCHNECK, M.D., MT. CARMEL, ILL.

The two following cases, while they may not present anything new, may yet be worth recording; as this form of urticaria is so cursorily referred to in most of our text-books. They do not call the attention of the reader sufficiently to this type of the disease to put him on his guard, and many, therefore, have to learn the disease by experience at the bedside, much to the annoyance of themselves and patient; at least, such was my experience.

CASE I.—July 25, 1873, at 11 A. M., was called, in great haste, to see Mr. A. S., a blacksmith, aged twenty; medium height, stout and robust. On my way there, the father, who came after me, gave me the following history of the case:

The patient had been as well as usual, until about six o'clock that morning, when he began to feel a tingling and itching sensation on the limbs and body, which, when rubbed or irritated, would break out with a profuse eruption of wheals. This became so annoying that he had to stop work and go home, where he was given warm tea, and both his mother and sister set to work rubbing, to bring out the eruption freely. While this was being done, he was taken with a severe convulsion.

Upon my arrival I found him in his third convulsion, which was a very severe one, and the body and limbs almost entirely covered with wheals. I immediately gave him thirty drops of chloroform, in a teaspoonful of water; and, as soon as the convulsive seizure was over, put him on compound spirit ether, one-half drachm every half hour, to prevent a return of the spasms.

Supposing the trouble to arise from some morbid accumulation in the stomach and bowels, I ordered a full dose of sulphate magnesia. Visited him again at 2 P. M. and found he had had no more convulsions, and was feeling well as usual, but "very tired." The magnesia had operated freely.

Heard no more of him until the 27th, about 11 A. M., when I was again called in great haste; found him in very much the same condition. Again resorted to Hoffman's Anodyne, with like success in stopping the paroxysms, he having but one more after my arrival. Suspecting that it might be of a periodic type, ordered quinine, twenty grains, to be divided into five powders, and given every two hours, on the morrow. There being no return of the trouble, I ordered the same amount of quinine to be taken once a week, for several weeks, to prevent a recurrence. Has had neither chill or convulsions since.

CASE II.—Was called April 7th, 1873, about 9 A. M., to see Mr. Wm. M., civil engineer, aged twenty-three; below medium size, but generally healthy. Found him freely broken out with wheals on all parts of the body; also with slight fever, burning and itching sensation of the skin, which seemed to be almost intolerable. Directed warm teas, and a seidlitz powder, to be repeated if necessary. Heard no more of him until the 9th, when I was again sent for, about 9 A.M.; was told that, in a few hours after my visit on the 7th, the eruption passed away, but came on that morning at the same time of day as on the 7th; found him in very much the same condition as two days previous. Ordered the warm teas again, and thinking it to be of the periodical type, quinine was ordered. On the next day the eruption again passed away in a few hours, and has never returned since.

In both cases, the patient attended to his usual avocations on the well day.

In neither case could I find a cause in the ingesta, over-exertion, or a delicate skin.

